# Deterministic Spin-Orbit Torque Induced Magnetization Reversal In Pt/[Co/Ni]_*n*_/Co/Ta Multilayer Hall Bars

**DOI:** 10.1038/s41598-017-01079-7

**Published:** 2017-04-20

**Authors:** Sihua Li, Sarjoosing Goolaup, Jaesuk Kwon, Feilong Luo, Weiliang Gan, Wen Siang Lew

**Affiliations:** School of Physical and Mathematical Sciences, Nanyang Technological University, 21 Nanyang Link, Singapore, 637371 Singapore

## Abstract

Spin-orbit torque (SOT) induced by electric current has attracted extensive attention as an efficient method of controlling the magnetization in nanomagnetic structures. SOT-induced magnetization reversal is usually achieved with the aid of an in-plane bias magnetic field. In this paper, we show that by selecting a film stack with weak out-of-plane magnetic anisotropy, field-free SOT-induced switching can be achieved in micron sized multilayers. Using direct current, deterministic bipolar magnetization reversal is obtained in Pt/[Co/Ni]_2_/Co/Ta structures. Kerr imaging reveals that the SOT-induced magnetization switching process is completed *via* the nucleation of reverse domain and propagation of domain wall in the system.

## Introduction

Control of magnetization dynamics using charge current is of great interest for applications in low power non-volatile memory, logic and neuromorphic devices^1–5^. The spin transfer torque (STT) effect from spin polarized current has been used to induce magnetization reversal in nano scale spintronics devices^6, 7^. STT arises mainly from spin-polarized electrons, produced by a ferromagnetic (FM) polarizer, transferring spin angular momentum to adjacent magnetic moments^8, 9^. Spin orbit torque (SOT) emerging from the material properties and interface between different layers has been reported as one of the promising candidates for magnetization switching governed by current^10–12^. In materials, spin-orbit coupling and broken symmetry can generate current-induced SOTs, which are able to stabilize the spin configuration in magnetic structures. As compared to STT, manipulating magnetization with SOT effect has been observed intuitively *via* current flow without the spin polarizer. SOT can be used for various metallic spintronics devices, due to the lower current density needed to reverse magnetic moment of ferromagnets^12, 13^.

Spin-orbit torque arises from the Spin-Hall effect (SHE) and Rashba effect in ultrathin heterostructures comprising of a ferromagnet (FM) in contact with heavy metal (HM) layers^14–17^. Charge current flowing in the HM generates spin current into the FM *via* SHE, whereas the spin-orbit interaction at the FM/HM interface induces spin accumulation by Rashba effect^16–19^. The collective actions of the spin current and spin accumulation result in a torque (SOT) on the local magnetization within the FM layer. The SOT-induced switching phenomenon^20–25^ is capable for free layer control in Magnetoresistive Random-Access Memory (MRAM), as only an in-plane charge current flow along constructed HM plain is necessary. To realize SOT-induced bipolar magnetization switching in the structure embedded perpendicular magnetic anisotropy (PMA) layer, an external in-plane magnetic field is required to break the symmetry along the current direction^26–28^. From a device standpoint, this is not ideal as the limitation of in-plane field in applications influences its integration and scaling.

Numerous techniques have been proposed to execute the field-free SOT-mediated magnetization switching. Lateral structural asymmetries introduced in wedged FM/HM structures were shown to produce a SOT-like switching wihout the assistance of external field^29, 30^. Tilted magnetic anisotropy adjusted from a wedged FM layer also resulted in bipolar switching of the ferromagnet^31^. More recently, antiferromagnetic (AFM) layers have been incorporated in FM/HM stack as an alternative to the structural modulation. Different stack configurations were investigated using a combination of IrMn and PtMn as AFM layer^32–34^. The in-plane exchange bias field induced by the AFM layer breaks the symmetry ^32, 33^ and induces a tilt in the out-of-plane magnetization of the FM layer. The AFM layer can substitute as an HM layer in the structure^32^. Another approach is to pin an in-plane FM (iFM) layer with an AFM layer^34^. The structure utilizes that the stray field from the iFM layer affects the magnetization of the FM layer to be switched. The basis of all these techniques is to provide an external stimuli so as to induce an out-of-plane tilt in the magnetization.

In this report, we show that by selecting PMA material with weak out-of-plane magnetic anisotropy, bipolar SOT-induced magnetization reversal can be achieved without the assistance of external magnetic field. Our material stacks comprise of Pt/[Co/Ni]_*n*_/Co/Ta thin films, where *n* = 2, 3 and 4. Bidirectional magnetization switching in the patterned micro Hall bars is observed with an in-plane DC current flow in the structure with *n* = 2. Kerr-microscopy images reveal that the reversal process is completed *via* nucleation and propagation of a domain wall (DW) in the path of current within the cross structure, which is large enough for domain nucleation and extension mechanism. Due to the intrinsic tilt of the magnetization, the nucleated DW adopts a chiral DW configuration during the reversal process.

## Results

### Device structure

**Figure 1:**
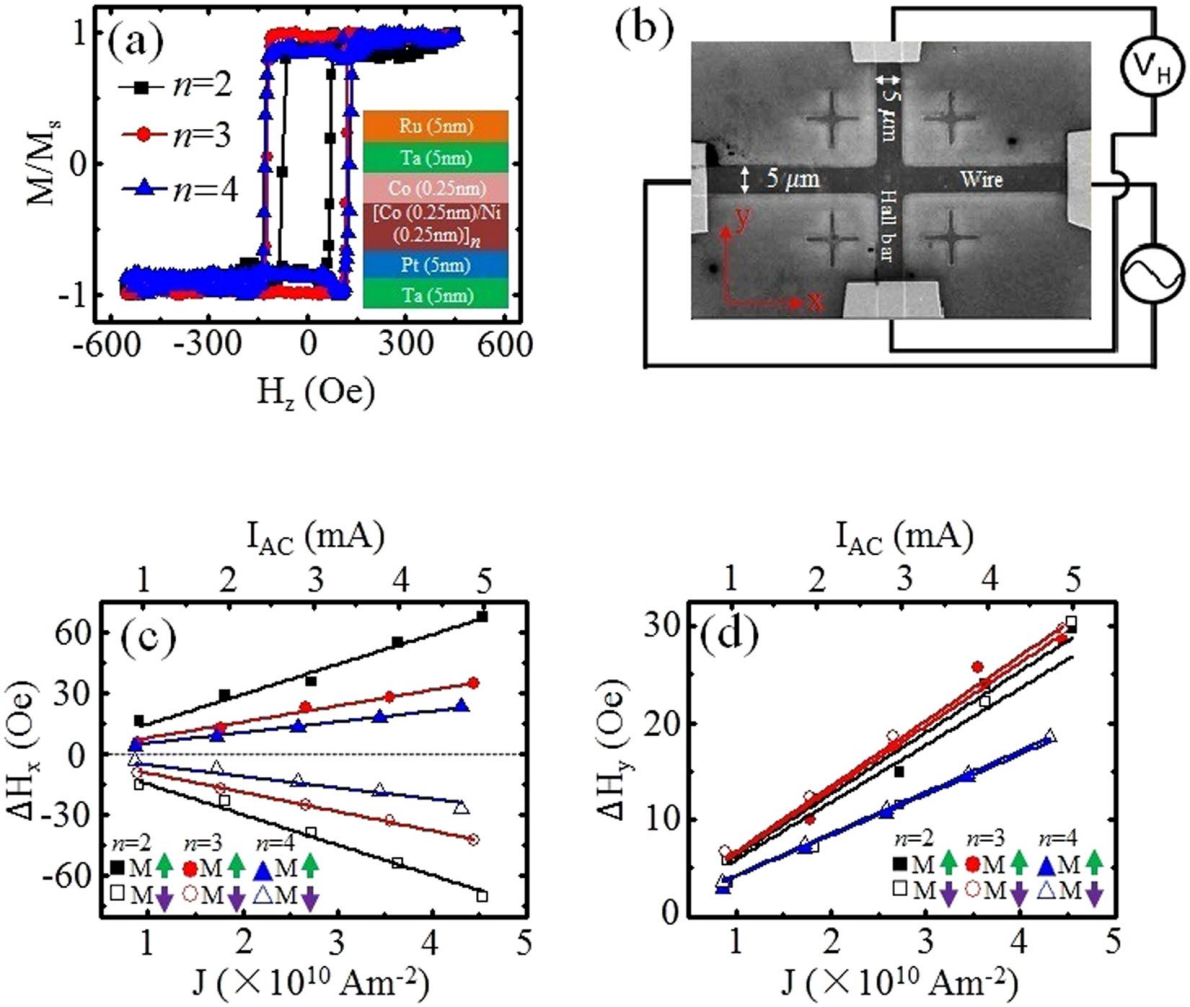
SOT effective fields in Pt/[Co/Ni]_*n*_/Co/Ta structures: (**a**) Normalized magnetization hysteresis loops measured using polar MOKE magnetometer. Inset shows schematic of Pt/[Co/Ni]_*n*_/Co/Ta thin film stack structures with *n* = 2, 3 and 4. All film stacks exhibit perpendicular magnetic anisotropy. (**b**) Scanning electron microscopy of fabricated cross-shaped structure with illustrated electrical measurement setup. AC current is applied along the wire, and a lock-in amplifier is used to measure the harmonic voltages across the Hall bar. (**c**) Damping-like effective fields of each sample for +*z* and −*z* magnetized states as a function of current density. (**d**) Field-like effective fields for each sample for +*z* and −*z* magnetized states as a function of current density.

Stacks comprising of Ta(5 nm)/Pt(5 nm)/[Co(0.25 nm)/Ni(0.5 nm)]_*n*_/Co(0.25 nm)/Ta(5 nm)/Ru(5 nm) films, where *n* = 2 (bilayer), 3 (trilayer) or 4 (four-layer), were grown using magnetron sputtering deposition technique. Normalized magnetization hysteresis loops were obtained using polar magneto-optical Kerr effect (MOKE) measurement technique, as shown in Fig. 1(a). The complete film stack structure is illustrated in the inset of Fig. 1(a). The coercivity of the bilayer film is 73.5 Oe. A large increase in the film coercivity to 121.7 Oe is observed when *n* increases to 3. For *n* = 4, the coercivity is almost the same as the trilayer. This is consistent with previous reports of coercivity change with the number layers of Co/Ni^35^. All film stacks exhibit clear perpendicular magnetic anisotropy as evidenced by the square hysteresis loops with sharp switching. The anisotropy energy density *K*_*u*_ is given by equation ${K}_{u}=\frac{1}{2}{M}_{S}\bullet {H}_{S}$. The saturation magnetization *M*_*S*_ and the hard axis saturation magnetic field *H*_*S*_ were measured by VSM (Vibrating Sample Magnetometer) technique. The corresponding values are: *M*_*S*_ = 539 emu/cm^3^ and 609 emu/cm^3^ for *n* = 2 and 4; *H*_*S*_ = 924.4 Oe and 4715.6 Oe for *n* = 2 and 4, respectively. The out-of-plane anisotropy energy density was calculated to be 2.5 × 10^5^ erg/cm^2^ for *n* = 2, increasing to 1.4 × 10^6^ erg/cm^2^ for *n* = 4, *via* the hard axis measurement method. The film stacks were patterned into cross-shaped structures, comprising of a wire and a orthogonal Hall bar, using a combination of electron beam lithography and Ar^+^ ion milling techniques. Figure 1(b) shows the scanning electron micrograph of the fabricated structure together with illustration depicting electrical measurement setup. The cross-shaped structure has a uniform width of 5 μm, while the lengths of the wire and Hall bar are 80 μm and 50 μm, respectively.

### SOT effective fields

Harmonic Hall voltage measurements^36^ were performed to quantify the SOT effective fields of the fabricated samples [see supplementary materials for measurement methodology]. Figure 1(c,d) show the damping-like and field-like effective fields of the different film stacks (*n* = 2, 3 and 4) for magnetization aligned along +*z* and −*z* orientations as a function of current density. A linear dependence of the effective fields on current density was obtained. The positive and negative damping-like effective fields correspond to the magnetization orientation with respect to the initial up (+*z*) and down (−*z*) magnetization states, respectively, as shown in Fig. 1(c). The field-like effective fields for initial up and down magnetized states have the same sign and amplitude as seen from Fig. 1(d). This is consistent with the field-like effective fields being independent of the magnetization orientation. The plots reveal a negative correlation between the FM layer thickness and the SOT effective fields. The bilayer sample exhibits much larger SOT fields as compared to the four-layer sample. For the bilayer sample, the damping-like effective fields increases by ~50 Oe as the current is increased from 1 mA to 5 mA, whereas for the four-layer sample, the increase is only ~20 Oe for the same current range.

### Current assisted magnetization switching

**Figure 2:**
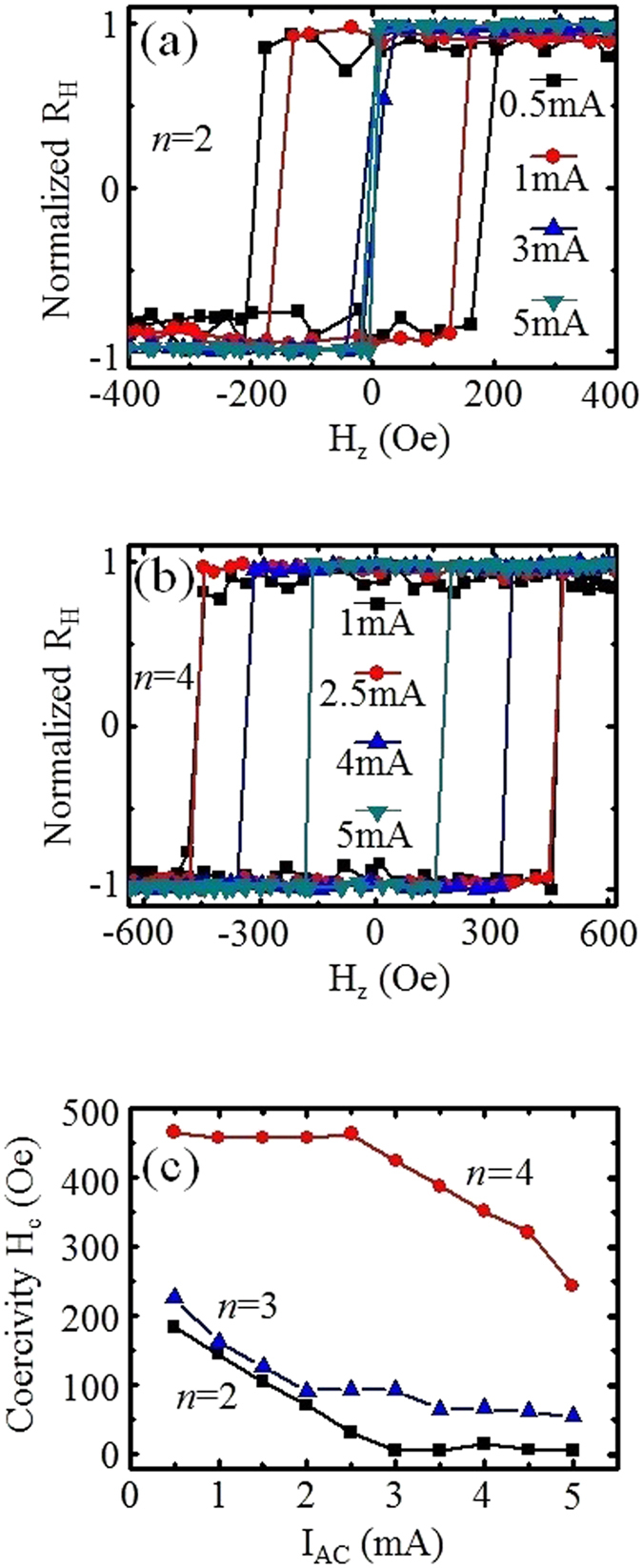
Representative AHE loops for different AC bias current measured by sweeping the external perpendicular magnetic field for both set of samples (**a**) *n* = 2 and (**b**) *n* = 4. (**c**) Measured coercivity as a function of the magnitude of the applied current for wire patterned with film stacks with *n* = 2, 3, and 4.

To investigate the SOT effect on the magnetization reversal process, anomalous Hall effect (AHE) measurements were conducted by sweeping an external out-of-plane magnetic field for different magnitude of AC bias current. Shown in Fig. 2(a,b) are the measured respective AHE loops for wires with film stack *n* = 2 and 4, respectively. For both structures, the switching field decreases as the bias current is increased. (The AHE loops for *n* = 3 are shown in supplementary Fig. S3). The corresponding switching fields extracted from the AHE loops, as a function of the AC bias current amplitude for stacked wire *n* = 2, 3 and 4, are presented in Fig. 2(c). For *n* = 2 and 3, the coercivity of the wire decreases linearly with respect to the applied current. A striking observation is that the coercivity of the stacked wire *n* = 2 is almost zero when the amplitude of the applied AC bias current is ≥3 mA. For the wire with *n* = 4, there is negligible change in the coercivity for current smaller than 3 mA. For current amplitude greater than 3 mA, the coercivity decreases linearly with current. The measurements were performed using a DC bias current on the structures with *n* = 2 structures and are presented in Fig. S4 of the supplementary information. In general, the coercivity decreases as the magnitude of the DC current increases and for I_DC_ >3 mA, the coercivity is almost zero. For DC current magnitude smaller than 3 mA, the positive and negative DC bias current lead to different coercivity trends. Similar change in coercivity as a function of DC current bias have been reported in structures comprising of HM/FM layer and the decrease in coercivity has been attributed to the dampling-like SOT fields induced by the current^32^. For all measurements, the current density in the wire was in the range of 10^10^ A/m^2^. To investigate the role of Joule heating on the coercivity trend, the wire resistance was monitored as a function of DC current bias. Though the variation of resistance at a fixed DC bias is ~0.1 Ω, a change of ~1 Ω was observed in the resistance of *n* = 2 sample as the current is increased from 1 mA to 3 mA. No similar change was observed for the *n* = 4 sample. The results are shown in Fig. S6. As such, the coercivity trend observed in our structures can be attributed to a combination of Joule heating and SOT-induced switching. We note that the coercivity decrease correlates with the corresponding measured SOT fields. The onset of coercivity change occurs when the damping-like SOT fields is greater than 15 Oe. This corresponds to a current of 0.5 mA for the bilayer sample and 3 mA for the four-layer sample. The coercivity of the bilayer sample reduces to zero for damping-like SOT field greater than 30 Oe.

**Figure 3:**
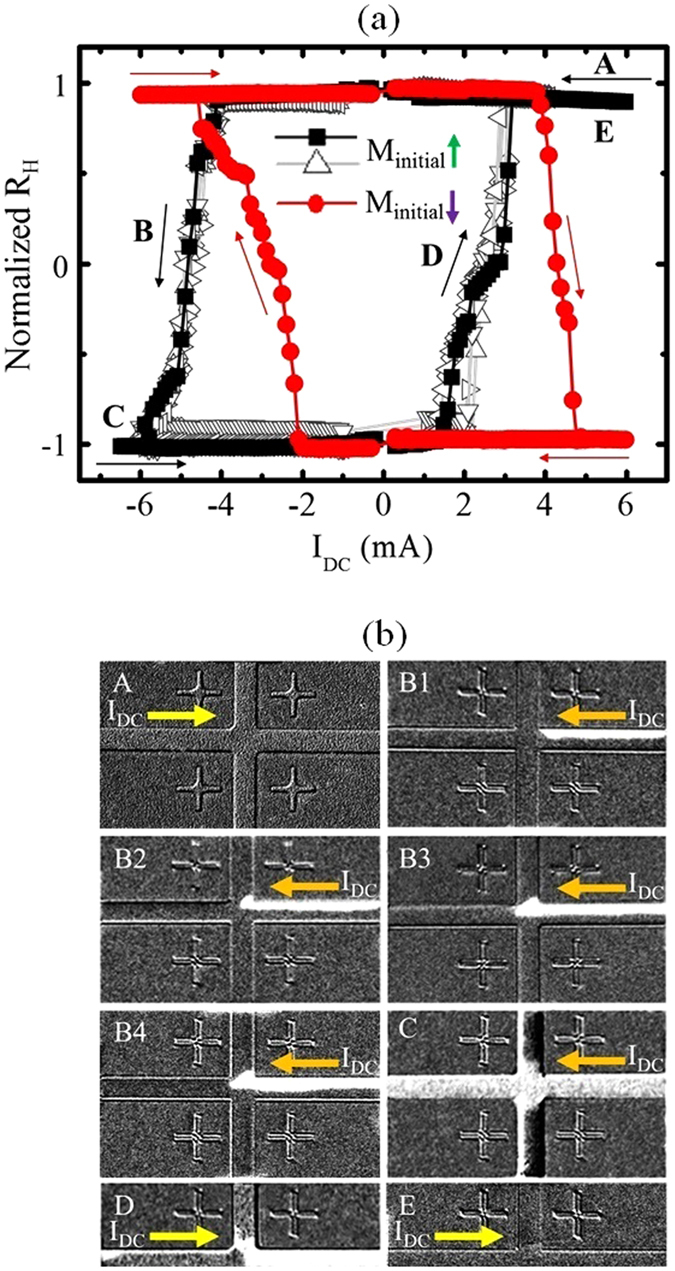
Field-free SOT-induced magnetization reversal process: (**a**) Measured R_H_ as a function of DC current without external magnetic field for Pt/[Co/Ni]_2_/Co/Ta structure. (**b**) Kerr images at different stages during the reversal process. Image A shows the initial up (+*z*) state; images B1–B4 show the gradual reversal process from up to down (−*z*) state; image C shows that the magnetization has completely switched to the down state; image D shows the quasi stable state as the magnetization is reversed in the opposite direction; image E shows that the magnetization has switched back to the up state again. The yellow arrows show the corresponding current directions.

To confirm the observed SOT switching, Hall resistance (R_H_) measurements with DC current sweeping were performed on the *n* = 2 structure, without any externally applied magnetic fields. Figure 3(a) shows the measured R_H_ as a function of the DC current. A clear hysteresis loop was obtained, implying a deterministic switching of the magnetization induced by the applied current. Firstly, the magnetization is aligned along the +*z* orientation. No change in R_H_ is observed when positive current (along +*x* direction) flows through the wire. As the current is swept in the opposite direction, negative current (along −*x* direction), R_H_ exhibits a step wise decrease at a current of −4 mA, reaching saturation at −6.5 mA. This implies that the magnetization has switched to the −*z* orientation. As the current is then increased towards +6.5 mA, the initial change in R_H_ signal occurs at 2 mA, with a quasi-stable state at +3 mA and saturation at +4 mA. This results in an asymmetric hysteresis loop, as shown by the black loop in Fig. 3(a). This asymmetric switching is due to an initial large current required to nucleate a reverse domain. The change in the Hall resistance corresponds to the domain crossing the Hall detector. Flowing current in the opposite direction is simply driving the reverse domain back along the Hall cross. When the initial magnetization is along the −*z* orientation, the switching to magnetization along +*z* orientation occurs at 4 mA. The switching from +*z* to −*z* occurs at a current of −2 mA, as shown by the red loop in Fig. 3(a). This implies that bi-directional current can be used to control the magnetization state of the wire. The loops with open symbols correspond to the different runs that were conducted with the initial magnetization states being along +*z* orientation and sweeping the DC current. For each run, a clear hysteretic loop is obtained, indicating that the switching process is deterministic in nature.

Direct observation of the magnetization switching process was carried out using quasi-static Kerr imaging technique. The sample was first saturated along the +*z* direction and the field was reduced to zero. This initial state was captured and used as a reference to be subtracted from subsequent images captured at different DC bias. Figure 3(b) shows the Kerr images at different instances during the switching process, corresponding to the states labelled in Fig. 3(a). The wire was initially saturated by a large magnetic field along the +*z* direction. The initial configuration, image A was captured after a positive current of +6.5 mA was applied to the wire. No change in magnetization configuration is observed. As the current was reduced from +6.5 mA to −4 mA, a reversed domain is nucleated in the wire, as evidenced by the bright contrast in Fig. 3(b)-B1, indicating the onset of magnetization switching. The magnetic configuration at the top half of the wire has flipped to the down (−z) state. Further increase of the current leads to the expansion of the reversed domain along both the wire length as seen in Fig. 3(b)-B2→B4. Complete magnetization reversal of the wire was achieved when the current was increased to −6.5 mA, as can be seen by the complete white contrast of the structure in Fig. 3(b)-C. As the current was increased from −6.5 mA to +2 mA, the quasi stable region in the Hall signal, Fig. 3(a), corresponds to the pinning of the DW at the Hall cross as seen in Fig. 3(b)-D. As the current was increased to positive saturation at +6.5 mA, the magnetization switched towards the up (+z) state again, as seen in Fig. 3(b)-E. The Kerr imaging confirms that the reversal process is completed *via* DW nucleation and propagation within the structure. The current can deterministically switch the magnetization orientation of the wire. Different orientations of current flow lead to different stable magnetization configurations.

Reversed domain nucleating from the edge of the wire has been reported by a combination of Oersted field and weakened anisotropy at the edges due to defects^37^. The out-of-plane component of the Oersted field has the maximum amplitude *H*_*z*−*Oersted*_ at the wire edges^38^. For a uniform strip conductor with width *w* and thickness *t*, *H*_*z*−*Oersted*_ can be approximated as ${H}_{z-Oersted}=Jt(3+2\,\mathrm{ln}(w/t))/4\pi$ when $t\ll w$ ^39^. In our geometry, the conductor can be considered as a 5 μm wide and 20 nm thick strip. The Oersted field is estimated to be ~0.84 mT for a curent of 3 mA (current density *J* = 1 × 10^10^ *Am* ^−2^), where the reversed domain is nucleated. This is much smaller than corresponding SOT fields at 3 mA which is ~30 Oe. To show that the combination of Oersted field and weakend anisotropy due to edge-defects do not play a role in the switching, the reversed process was repeated for initial magnetization along +*z* and −*z* directions. Figure S7 shows that though the reversal starts from the edge of the wire, the region where the reversed domain nucleates is different. For each instance, the reversed domains are nucleated closer to the contact where the current is applied. This implies that the Oersted field and edge-defects do not play a significant role in the reversal domain nucleation.

## Discussion

**Figure 4:**
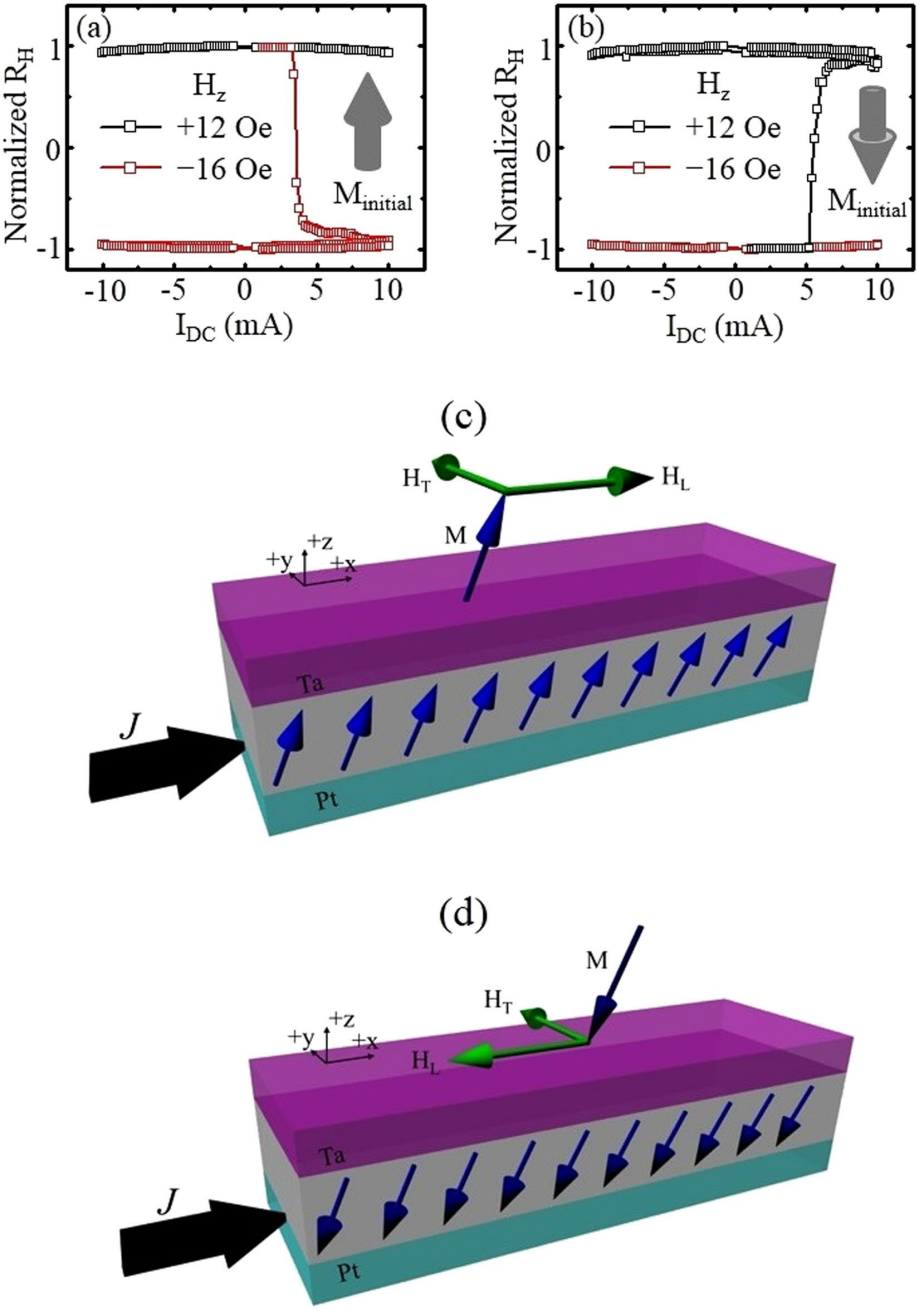
Tilting of the magnetization: Measured R_H_ as a function of the DC current with a small fixed out-of-plane magnetic field along the +*z* or −*z* directions for (**a**) up and (**b**) down initial magnetization states. (**c**) and (**d**) are the schematic magnetic configurations corresponding to (**a**) and (**b**), respectively.

The deterministic SOT switching is ascribed to a tilt in the out-of-plane magnetization induced by the combination of Joule heating and SOT in the bilayer structure. To experimentally assess this presumptive explanation, a small out-of-plane magnetic field was applied and the in-plane current sweeping measurements on samples with magnetization along the +*z* orientation was performed. When the out-of-plane field of 12 Oe was applied along the +*z* direction, no magnetization switching behavior was observed in the measured AHE signal, even for current strengthened up to 10 mA, as indicated by the black data line in Fig. 4(a). However, when the field (−16 Oe) direction was reversed, *i.e*. along the −*z* direction, which was also opposite to the magnetization orientation, a sharp change in the AHE signal was observed, implying the switching of the magnetization state, as shown by the red data line in Fig. 4(a). Once the magnetization switches, it was aligned with the applied field direction, and no further switching was observed even when the current direction was reversed. Similar behavior was observed when the initial magnetization of the device is set along the −*z* orientation, as shown in Fig. 4(b). These experimental results confirm our explanation that the marginal tilt of the magnetization with respect to the *z*-axis in the bilayer structure contributes to the field-free SOT reversal process. Figure 4(c,d) depict the configurations of current induced SOT fields for initial magnetization along +*z* and −*z* directions, corresponding to Fig. 4(a,b), respectively. The direction of the transverse effective field *H*_*T*_ is determined by $\hat{z}\times \vec{J}$, which is independent of the initial magnetization state. The direction of the longitudinal effective field *H*_*L*_ is determined by $\hat{m}\times \hat{s}$, where $\hat{m}$ is the magnetization direction and $\hat{s}$ represents the average spin direction of the electrons diffusing into the magnetic layer. In our configurations, the collective spin Hall effect from Pt underlayer and Ta capping layer results in a positive spin Hall angle, which aligns $\hat{s}$ pointing to $-(\hat{z}\times \vec{J})$. Therefore, the direction of the longitudinal field is determined by $-(\hat{m}\times (\hat{z}\times \vec{J}))$, which is dependent of the initial magnetization state, as shown in Fig. 4(c,d).

To gain an insight into the SOT-induced DW motion leading to the magnetization switching, R_H_ measurements were carried out by sweeping DC current with various fixed in-plane magnetic fields (*H*_*x*_) applied along the wire long axis. The measured R_AHE_ vs I_DC_ loops at different *H*_*x*_ are shown in Fig. S5 in supplementary. Irrespective of the magnitude of the negative *H*_*x*_, complete magnetization reversal at the Hall cross is obtained, as evidenced by the maximum change in R_H_ magnitude. For positive *H*_*x*_ less than 500 Oe, a smaller change in R_H_ magnitude is obtained, implying incomplete switching at the Hall cross. As the positive *H*_*x*_ is increased, the change of R_H_ in magnitude increases correspondingly, reaching the saturation for *H*_*x*_ ≥ 500 Oe. Our result is consistent with Pt being the dominant source of Dzyaloshinskii-Moriya interaction (DMI) and leading to a negative DMI effective field *H*_*DMI*_, stabilizing a left handed Néel DW.

**Figure 5:**
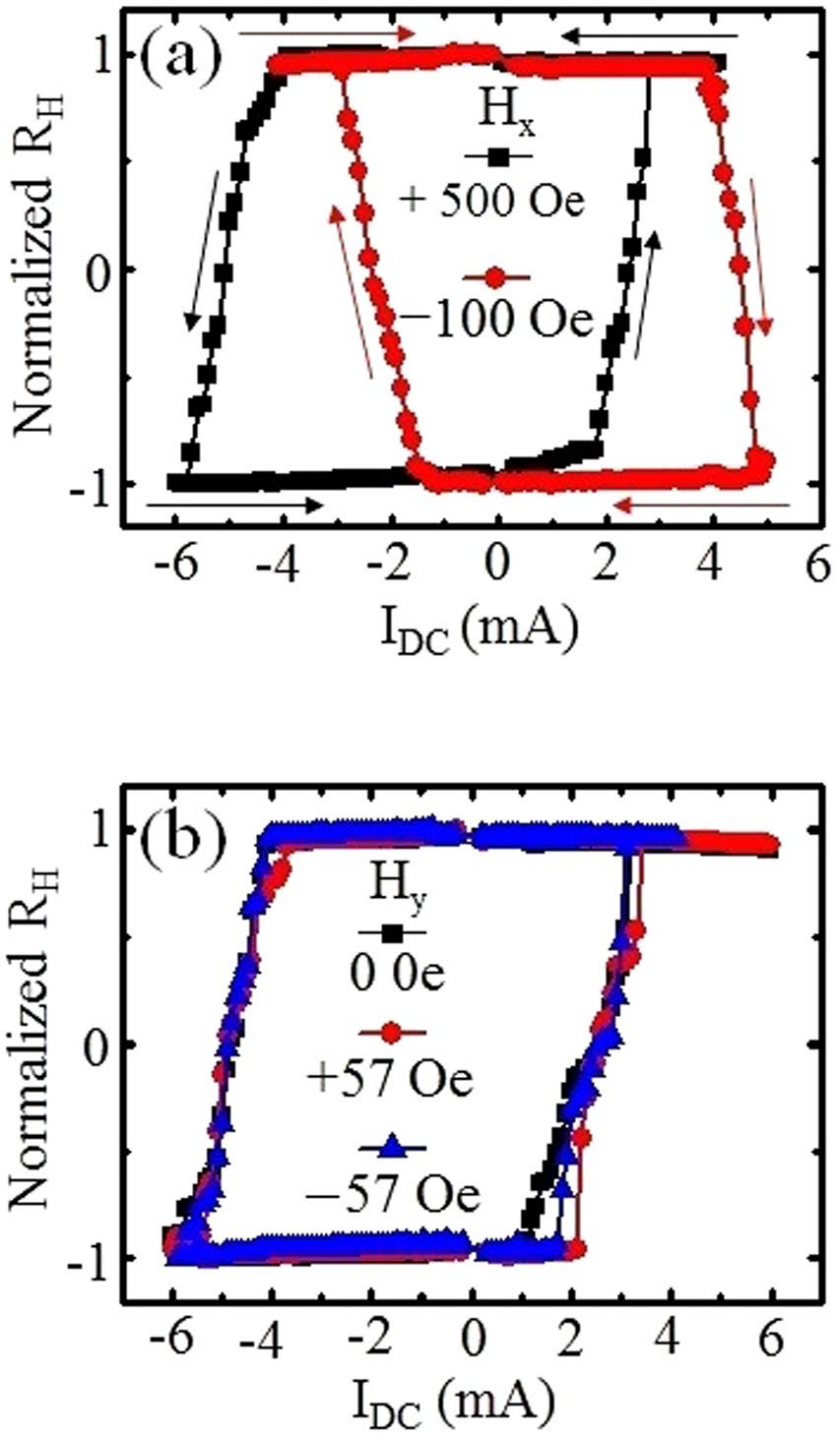
Current-induced magnetization switching with an in-plane external magnetic field: (**a**) R_H_-I loops measured by sweeping DC current with a fixed in-plane magnetic field along the (black) +*x* and (red) −*x* directions. (**b**) Measured R_H_-I loops with sweeping DC current when a fixed in-plane magnetic field was applied along the (red) +*y* and (blue) −*y* directions together with the loop for (black) zero field.

The representative loops for *H*_*x*_ = +500 Oe and −100 Oe are shown in Fig. 5(a). For the measured R_H_-I_DC_ loops with fields applied along the +*x* (+500 Oe) or −*x* (−100 Oe) orientations in Fig. 5(a), complete magnetization switching at the Hall cross is obtained. Despite both field orientations displaying clear hysteresis, the switching behavior is completely different. When a positive *H*_*x*_ is applied, a R_H_-I_DC_ loop similar to the result without any applied external field was obtained. However, when the applied *H*_*x*_ is along the −*x*-direction, the magnetization switching from +*z* to −*z* direction occurs when a positive current was applied. The discrepancy is attributed to the stabilization of different DW configurations with the application of *H*_*x*_. The direction of the *H*_*x*_ favors a particular handedness chirality of the DW mediating the reversal process. For *H*_*x*_ applied along the +*x* orientation more than +500 Oe, a right-handed Néel DW is obtained. It is known that for SOT-induced Néel DW motion in structure with Pt underlayer, the left-handed DW moves along the direction of current, whereas the right-handed DW moves in the opposite direction of current. This is consistent with the measured R_H_-I loops property shown in Fig. 5(a).

Similar measurements were conducted with an in-plane transverse field. Figure 5(b) shows the representative R_H_-I loops in the presence of the transverse field along the + or −*y*-directions. It is worth noting that the transverse field does not affect the switching behavior and the magnitude of current required to induce switching. Additionally, irrespective of the field orientation, the same switching behavior implies that the magnetization reversal process is mediated by the damping-like term of the SOT.

## Conclusion

In conclusion, field-free bipolar SOT-induced magnetization reversal can be achieved by a weak out-of-plane magnetic anisotropy in as grown Pt/[Co/Ni]_2_/Co/Ta structures. Kerr imaging reveals that the reversal process is completed *via* the nucleation and propagation of DW within the wire. Due to the intrinsic tilt of the magnetization, the nucleated DW adopts a specific DW configuration during the reversal process. These results paves the way for an alternative approach to induce field-free SOT-induced magnetization switching.

## Methods

### Sample preparation

The thin film stacks of Ta(5 nm)/Pt(5 nm)/[Co(0.25 nm)/Ni(0.5 nm)]_*n*_/Co(0.25 nm)/Ta(5 nm)/Ru(5 nm), where *n* = 2, 3 and 4, were deposited on thermally oxidized silicon substrates using DC magnetron sputtering deposition technique at room temperature. Polar magneto-optical Kerr effect (MOKE) measurement was used to characterize the magnetic properties of the thin film stack. The micro wire with an orthogonal Hall bar was fabricated using a combination of electron beam lithography and Ar^+^ ion milling techniques. The wire is 80 μm long and 5 μm wide and the Hall bar is 50 μm long and 5 μm wide. Ta(6 nm)/Cu(94 nm)/Au(24 nm) electrodes were also fabricated using electron beam lithography and lift-off following DC magnetron sputtering. Argon reverse sputtering was carried out before sputtering electrodes to obtain a better Ohmic contact.

### Harmonic Hall voltage measurement

The harmonic Hall measurements were conducted by sinusoidal current with amplitude varying from 1 mA to 5 mA using an AC/DC sourcemeter (Keithley 6221). A Lock-in amplifier (SIGNAL RECOVERY 7265) was used to detect the first and second harmonic voltages across the Hall bar. For all AC current measurements, the frequency was fixed at 333 Hz.

### Hall resistance measurement

The Hall resistance (R_H_) measurements were carried out on a Cascade Microtech probe station. The Hall resistances were determined by measuring the voltage between the Hall bar using a multimeter (Keithley 2000) while sweeping a DC current from −6.5 mA to +6.5 mA along the wire using a sourcemeter (Keithley 2400). All measurements were performed at room temperature.

## Electronic supplementary material

SUPPLEMENTARY MATERIALS
