# Supplementary material for: Deterministic Spin-Orbit Torque Induced Magnetization Reversal In Pt/[Co/Ni]n/Co/Ta Multilayer Hall Bars
Source: Sci Rep. 2017 Apr 20;7:972. doi: 10.1038/s41598-017-01079-7 (PMC5430536; doi:10.1038/s41598-017-01079-7)
Supplement: Supplementary file 1 — SUPPLEMENTARY MATERIALS [file 41598_2017_1079_MOESM1_ESM.pdf]

SUPPLEMENTARY MATERIALS

Deterministic Spin Orbit Torque Induced Magnetization Reversal in Pt/[Co/Ni]$_n$/Co/Ta Multilayer Hall Bars

Sihua Li, Sarjoosing Goolaup, Jaesuk Kwon, Feilong Luo, Weiliang Gan and Wen Siang Lew

School of Physical and Mathematical Sciences, Nanyang Technological University, 21 Nanyang Link, Singapore 637371

Table of Contents:

S1. Harmonic Hall voltage measurement technique

S2. Harmonic Hall voltage signals

S3. The contribution of SHE to SOT

S4. AHE measurement using AC bias current for *n* = 3 structures

S5. AHE measurements using DC bias current for *n* = 2 structures

S6. Current induced switching with longitudinal external magnetic fields

S7. Joule heating effect on the coercivity trend

S8. Effect of Oersted field on reversed domain nucleation

S1. Harmonic Hall voltage measurement technique

For the purpose of characterizing the magnitude and direction of the effective fields induced by SOT, the illustration of the harmonic Hall voltage measurement scheme for a ferromagnetic heterostructure system with perpendicular magnetic anisotropy (PMA) is shown in Fig. S1. The magnetic energy density of the system can be expressed as [1-3]

$$E_{total} = -K_{eff}cos^2\theta - K_{in}sin^2\theta\, sin^2\varphi - \boldsymbol{M}_s \cdot \boldsymbol{H}, \tag{1}$$

where the effective perpendicular anisotropy energy is $K_{eff} = K_u - \frac{1}{2}(N_z - N_x)\mu_0 M_s^2$. K_u is the uniaxial perpendicular magnetic anisotropy energy and is a positive value for out of plane magnetic easy axis (z-axis). $\boldsymbol{M}_s$ is the saturation magnetization, and the in-plane anisotropy energy $K_{in} = \frac{1}{2}(N_x - N_y)\mu_0 M_s^2$ is from the shape anisotropy of the nanowire. In this system, the demagnetization factors along basic directions satisfy $N_z \gg N_y > N_x$ due to the geometry of the wire. The in-plane easy axis in our system is along the x-axis (the wire direction). Hence, we neglect N_x and N_y , and set $N_z = 1$ for the convenience of computation.

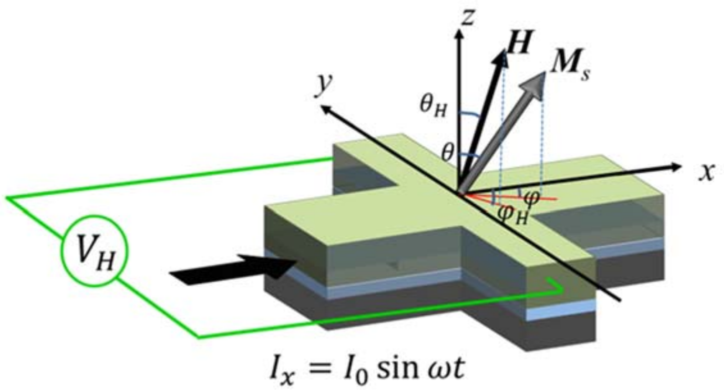

Figure S1. The schematic illustration of the coordinate system. $\boldsymbol{M}_s$ and $\boldsymbol{H}$ represent the magnetization and the external magnetic field respectively. θ and φ are the polar and azimuthal angles of the magnetization respectively. θ_H and φ_H are the polar and azimuthal angles of the external magnetic field respectively.

Using the extraordinary Hall effect (EHE), the Hall voltage V_{xy} is a product of the Hall resistance R_{xy} and the applied current I_x, expressed as[4]

$$\mathrm{V}_{xy} = R_{xy} I_x = \frac{\Delta R_H}{2} I_x \cos\theta. \quad (2)$$

Considering an AC current $I_x = I_0 sin\omega t$ applied, the polar and azimuthal angles of the magnetization will oscillate as $sin\omega t$, expressed as $\theta = \theta_0 + \Delta\theta sin\omega t$ and $\varphi = \varphi_0 + \Delta\varphi sin\omega t$. $\Delta\theta$ and $\Delta\varphi$ are the modulation amplitudes of the magnetization angle from its equilibrium position (θ_0, φ_0). The Hall voltage V_{xy} can be expressed in harmonic form as

$$V_{xy} = V_0 + V_\omega sin\omega t + V_{2\omega} sin2\omega t + \cdots, \quad (3)$$

Where, V_ω and $V_{2\omega}$ are the first and second harmonic voltage, respectively.

For our system, the initial magnetization $\boldsymbol{M}$ lies perpendicular to the film plane due to a high K_{eff}. As we apply an external out-of-plane magnetic field H_z along the z direction, we obtain

$$V_\omega \approx \pm\frac{1}{2}\Delta R_H I_0. \quad (4)$$

The ± sign corresponds to the case for up ($\boldsymbol{M}_s$ along +**z** direction) and down ($\boldsymbol{M}_s$ along -**z** direction) magnetization states, respectively.

For the case of an external in-plane magnetic field, $\theta_H = \frac{\pi}{2}$, both the first and second harmonic Hall voltage can be obtained by

$$V_{\omega,(x,y)} \approx \pm\frac{1}{2}\Delta R_H I_0 (1 - \frac{H_{(x,y)}}{2H_K})^2 , \quad (5)$$

$$V_{2\omega,(x,y)} \approx -(\pm\frac{1}{4}\Delta R_H I_0)\Delta H_{(x,y)} \frac{H_{(x,y)}}{H_K^2}. \quad (6)$$

Here, H_K is defined by $H_K = \frac{2K_{eff}}{M_s}$. H_x and H_y represent the longitudinal and transverse external magnetic field, respectively. The ± sign corresponds to the case for up and down magnetization states, respectively.

The effective fields are obtained by:

$$\Delta H_{(x,y)} = -2\frac{\partial V_{2\omega}}{\partial H_{(x,y)}} / \frac{\partial^2 V_{\omega}}{\partial H_{(x,y)}^2}. \quad (7)$$

S2. Harmonic Hall voltage signals

The harmonic Hall voltage measurements were conducted to detect the first harmonic voltage V_{ω} and second harmonic voltage $V_{2\omega}$ of the Hall bar with a constant amplitude sinusoidal current $I_x = I_0 sin\omega t$. Figures S2(a)-(e) show the experimental results. Figure S2(a) shows the anomalous Hall effect result measured with an AC current amplitude of $I_0 = 5mA$, where the perpendicular external magnetic field H_z sweeping field from ~–750 Oe to ~+750 Oe was applied. The change of AHE is about 0.9 mV. ΔR_H is calculated to be $\Delta R_H = \frac{0.9mV}{5mA} = 0.18\Omega$, according to eq. (4). Figure S2(b) shows the first harmonic Hall voltage with longitudinal external in-plane magnetic field H_x in the range of ~–750 Oe - ~+750 Oe for up and down magnetization, respectively. Figure S2(c) shows the first harmonic Hall voltage with transverse external in-plane magnetic field H_y in the same range for up and down magnetization, respectively. These two results are both fitted by eq. (5) of first harmonic Hall voltage, respectively. The curvatures for up and down magnetization are of a similar value. Figure S2(d) shows the second harmonic Hall voltage with longitudinal external in-plane magnetic field H_x in the range of ~–750 Oe - ~+750 Oe for up and down magnetization, respectively. Figure S2(e) shows the second harmonic voltage with transverse external in-plane magnetic field H_y in the same range for up and down magnetization. These two results are both fitted by eq. (6) of second harmonic Hall voltage, respectively. For the longitudinal results, the slopes of the fitting curves of up and down magnetization are also of a similar value. While for the transverse fields, the slopes of the fitting curve of up and down magnetization are of a similar value but opposite signs. According to eq. (7), we calculate the

longitudinal effective fields ΔH_x = –23 Oe and 21 Oe for up and down magnetization, respectively, and the transverse effective fields ΔH_y = 19.80 Oe and 19.20 Oe for up and down magnetization, respectively.

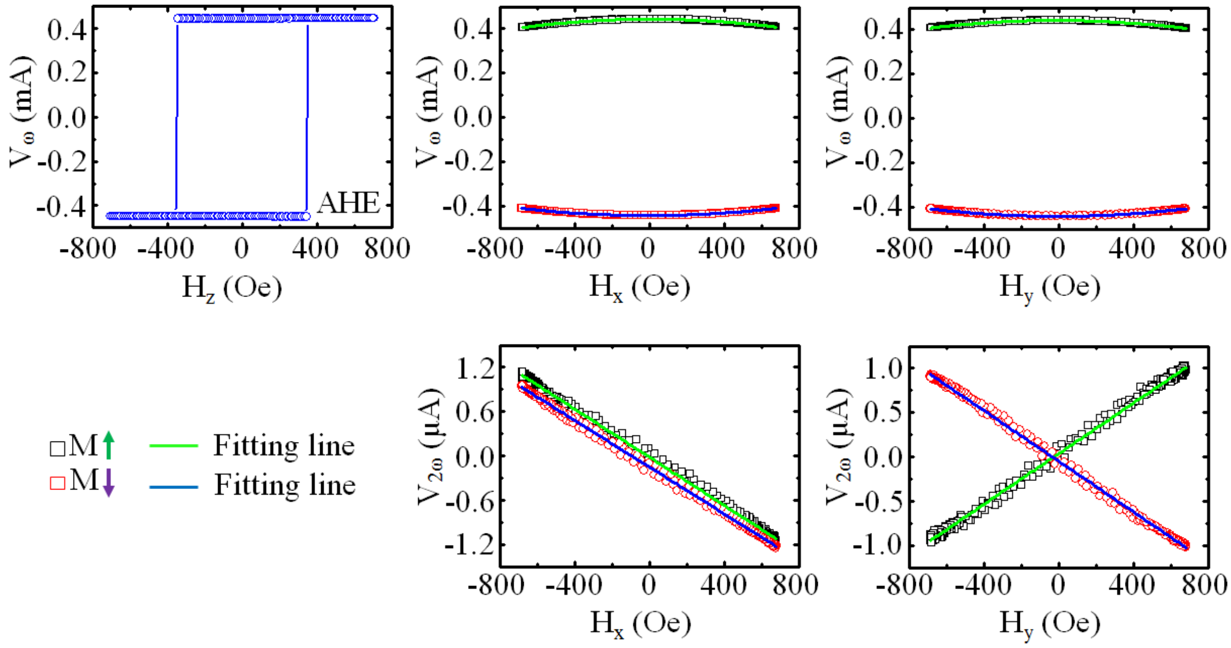

Figure S2. Harmonic Hall voltage signals. (a) The anomalous Hall effect (AHE) hysteresis loop by AC measurement with perpendicular external magnetic field (H_z). (b) First Harmonic signals with longitudinal external magnetic field (H_x) for up and down magnetization. (c) First Harmonic signals with transverse external magnetic field (H_y) for up and down magnetization. (d) Second harmonic signals with longitudinal external magnetic field (H_x) for up and down magnetization. (e) Second harmonic signals with transverse external magnetic field (H_y) for up and down magnetization.

To investigate current density and Co/Ni layer numbers dependence of the SOT, the same harmonic Hall voltage measurements were conducted for all the samples with various current densities. The comparison of the longitudinal ΔH_x and transverse ΔH_y effective fields per unit current density for the samples is shown in Table S1, which shows the negative correlation between the effective fields and the Co/Ni layer numbers.

Table S1. The comparison of the longitudinal ΔH_x and transverse ΔH_y effective fields per unit current density for n=2 and 4.

Sample	*n*=2	*n*=4
ΔH_x (Oe/10^{10}Am^{-2})	14.8	5.4
ΔH_y (Oe/10^{10}Am^{-2})	6.1	4.3

S3. The contribution of SHE to SOT

The SOT effective fields in Co/Ni multilayer systems show a linear relationship as a function of the applied current. The direction of the longitudinal effective fields (damping-like term) depends on the magnetization direction, while the direction of the transverse effective fields (field-like term) is independent of the magnetization direction, corresponding to damping-like term and field-like term, respectively. Both the longitudinal and transverse effective fields decrease as Co/Ni layer number increases. The longitudinal effective fields are determined by the saturation magnetization M_s and the thickness of ferromagnetic layer thickness t_{FM}, expressed as $\Delta H_x / J = \frac{\alpha_x}{M_s t_{FM}}$ [1], where $\alpha_x = \frac{\alpha_H \hbar}{2e}$ is a constant. The measured values of M_s are 539 emu/cm^3 for *n*=2 and 609 emu/cm^3 for *n*=4. The values of t_{FM} are 1.75 nm for *n*=2 sample and 3.25 nm for *n*=4 sample. From the equation, the ratio of the longitudinal effective fields for *n*=2 and *n*=4 is $\delta_x = \frac{(\Delta H_x / J)_{n=2}}{(\Delta H_x / J)_{n=4}} = \frac{(M_s)_{n=4} \bullet (t_{FM})_{n=4}}{(M_s)_{n=2} \bullet (t_{FM})_{n=2}} = 2.1$.

As shown in Table 1, the longitudinal effective fields per current density are 14.8 Oe/10^{10}Am^{-2} for *n*=2 and 5.4 Oe/10^{10}Am^{-2} for *n*=4. The measured value of the ratio is $\delta_x = \frac{(\Delta H_x / J)_{n=2}}{(\Delta H_x / J)_{n=4}} = \frac{14.8}{5.4} = 2.7$. The difference between the theoretical and experimental ratio

values of δ_x is $\frac{2.7-2.1}{2.7} \approx 22\%$. This is consistent with the ~10% decrease in the current flowing in respective top and bottom heavy metal layer (Ta and Pt) considering spin Hall effect (SHE), effectively resulting in a ~20% decrease in the charge current flowing through the HM layers. The transverse effective fields are determined just by the saturation magnetization M_s, according to equation $\Delta H_y / J = \frac{\alpha_y}{M_s}$[5,6], where $\alpha_y = \frac{\alpha_R P_{sd}}{\mu_B}$. Similarly, the value of ratio is $\delta_y = \frac{(\Delta H_y / J)_{n=2}}{(\Delta H_y / J)_{n=4}} = \frac{(M_s)_{n=4}}{(M_s)_{n=2}} = 1.1$. As shown in Table 1, the transverse effective fields per current density are 6.1 Oe/10^{10}Am^{-2} for n=2 and 4.3 Oe/10^{10}Am^{-2} for n=4. The measured value of the ratio is $\delta_y = \frac{(\Delta H_y / J)_{n=2}}{(\Delta H_y / J)_{n=4}} = \frac{6.1}{4.3} = 1.4$. The difference between the theoretical and experimental ratio values of δ_y being $\frac{1.4-1.1}{1.4} \approx 21\%$ is also found to be in accordance with the ~20% decrease in current top and bottom heavy metal layers. This suggests that Rashba effect is not the main contributor of the transverse effective field. Hence we conclude that the SHE contributes to both the longitudinal and transverse effective fields. In our stack structures, we assume that the spin Hall effect arises from the two heavy metals, Pt and Ta, sandwiching the FM layer. As Pt and Ta have spin Hall angles of opposing signs, the collective spin orbit torque induced by the respective layers should be constructive in nature, leading to an enhanced SOT within the FM layer.

S4. AHE measurements using AC bias current for *n* = 3 structures

AHE measurement was conducted by sweeping an external out-of-plane magnetic field for different magnitude of AC bias current. Shown in Figs. S3 is the measured AHE loops for n = 3 structures. The switching field decreases as the bias current is increased. The

corresponding switching fields extracted from the AHE loops, as a function of the AC bias current amplitude are presented in Fig. 2(c) in the manuscript. The value of coercivity H_c is given by $H_c = \frac{(H_{c+} - H_{c-})}{2}$, where H_{c+} and H_{c-} are positive and negative switching fields, respectively.

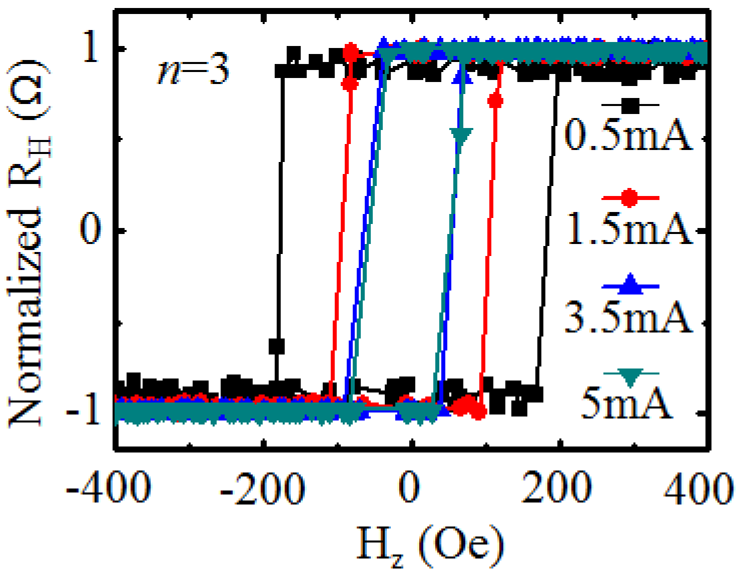

Figure S3. AHE loops for different AC bias current measured by sweeping the external perpendicular magnetic field for n = 3 structures.

S5. AHE measurements using DC bias current for *n* = 2 structures

For all the AC current measurements, the frequency was set to 333 Hz while the AC current values refer to the amplitude. Given that the AHE measurements were conducted for field induced reversal, the AC current effect is only to aid in the reversal process, although the AC current induces an oscillating SOT within the system.

AHE measurements have also been performed using DC bias current on n = 2 structures. Both positive (along +x orientation) and negative (along –x orientation) current were used. The measured AHE loops by various current values are shown in Figs. S4(a) and (b),

respectively. The switching field is decreased as current value increases. The coercivity values extracted from corresponding AHE loops including DC and AC measurements were plotted in the Fig. S4(c). In the same method, the value of coercivity H_c is given by $H_c = \frac{(H_{c+} - H_{c-})}{2}$, where H_{c+} and H_{c-} are positive and negative switching fields, respectively.

The positive and negative DC bias currents lead to different coercivity trends for current smaller than 3mA, as seen in Fig. S4(c). This change in coercivity cannot be explained by just Joule heating effect, which is dependent on the magnitude of the current but independent of the current directions.

Interestingly, for small current, irrespective of the DC current direction, the obtained coercivity is much lower that for AC current. This is consistent with the lower RMS value ($I_{AC}/\sqrt{2}$) of the AC current. However, for current larger that 3mA, we note that the current induced magnetization reversal dominates irrespective of the type of current bias used.

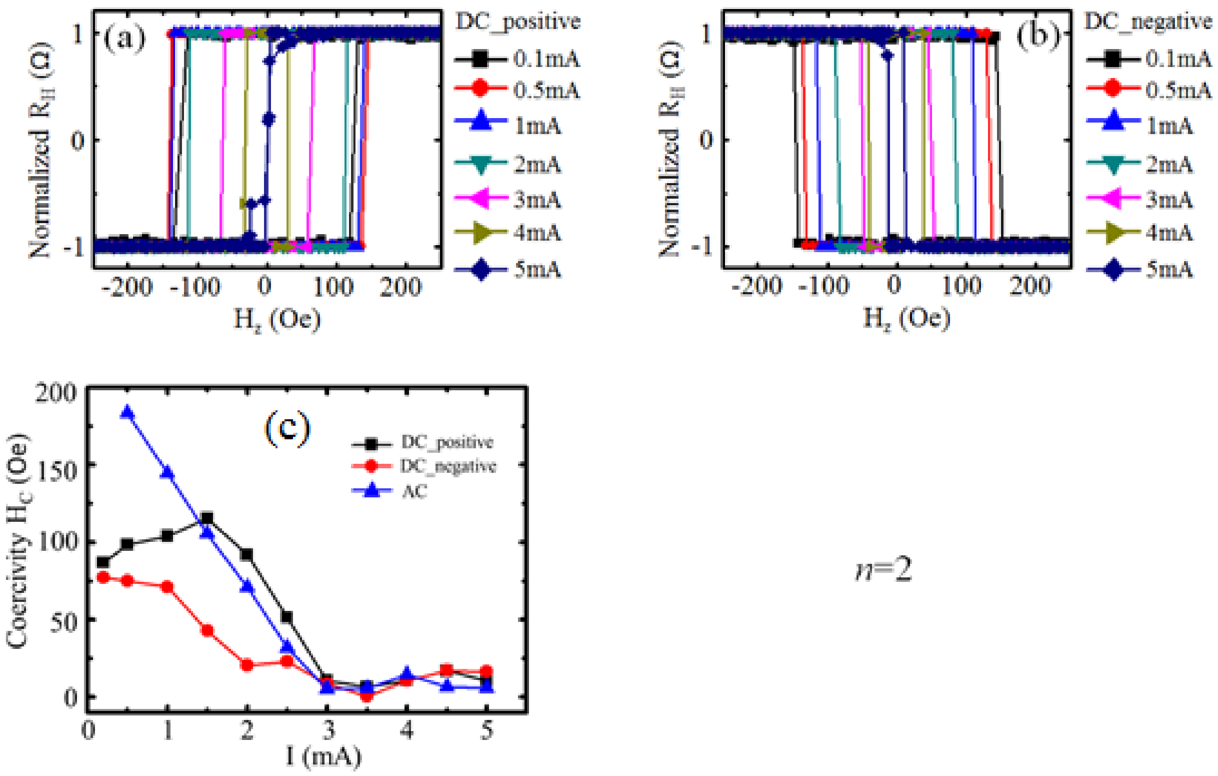

n=2

Figure S4. AHE loops measured by positive (a) and negative (b) DC current for n = 3 structures. (c) Coercivity trends on DC and AC current.

S6. Current induced switching with longitudinal external magnetic fields

R_H measurements by sweeping DC current were carried out with various fixed in-plane magnetic field applied along the wire long axis. The measured R_{AHE} vs I_{DC} loops at different H_x are shown in revised Fig.S5. The two figures show the change of the Hall resistance by sweeping DC current with an applied longitudinal external magnetic field along (a) +x and (b) –x directions. For the negative fields, different magnetic fields result in similar R_{AHE} loop. For the positive fields, different magnetic fields lead to different magnitude in the Hall resistance, whereas the switching occurs at almost similar current. The change in magnitude for the AHE loop corresponds to the magnetization switching at the junction. A lower magnitude implies that

a smaller region has changed at the Hall cross.

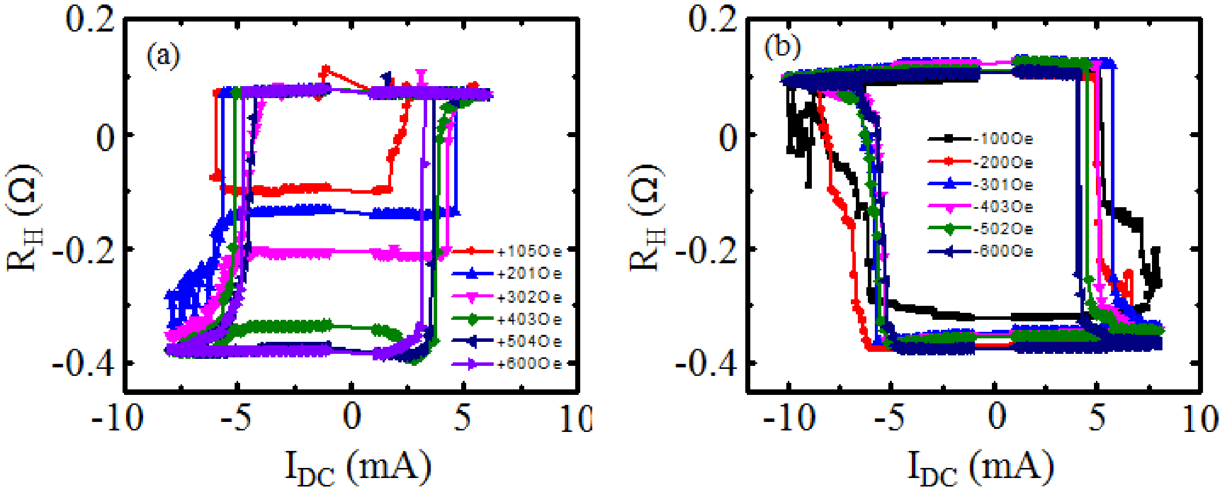

Figure S5. The R_H vs I_{DC} loops with different applied longitudinal external magnetic fields along (a) +x and (b) –x directions.

Figure S5 infers that the negative filed can result in the full reversal at the Hall cross evidenced by the maximum change in R_H magnitude. Whereas for the positive filed, the full switching at the Hall cross only occurs when the filed is larger than 500 Oe. For the positive filed smaller than 500 Oe, the Hall cross can be reversed only partially evidenced by the lower change in R_H magnitude. Therefore, the DMI effective field H_{DMI} in our sample structure is negative, acting along the $-x$ orientation. Negative H_{DMI} results in a left handed Néel DW chirality. This is consistent with Pt being the dominant source of DMI.

S7. Joule heating effect on the coercivity trend

To assess the Joule heating effect, the resistance of the wire was monitored by a DC current flow for 10 minutes. Figure S6(a) shows the measured wire resistance for $n = 2$ structures by 1mA and 3 mA, respectively. Though the variation of resistance at a fixed DC bias is ~0.1 Ω, a change of ~1 Ω was observed in the resistance of $n = 2$ sample as the current is increased from 1 mA to 3 mA, where the coercivity is tuned by current. Therefore, the Joule heating assists on the reversal process. No similar change was observed for the $n = 4$ sample, as shown in Fig. S6(b). As such, the coercivity trend observed for $n = 2$ structures can be attributed to a combination of joule heating and SOT-induced switching.

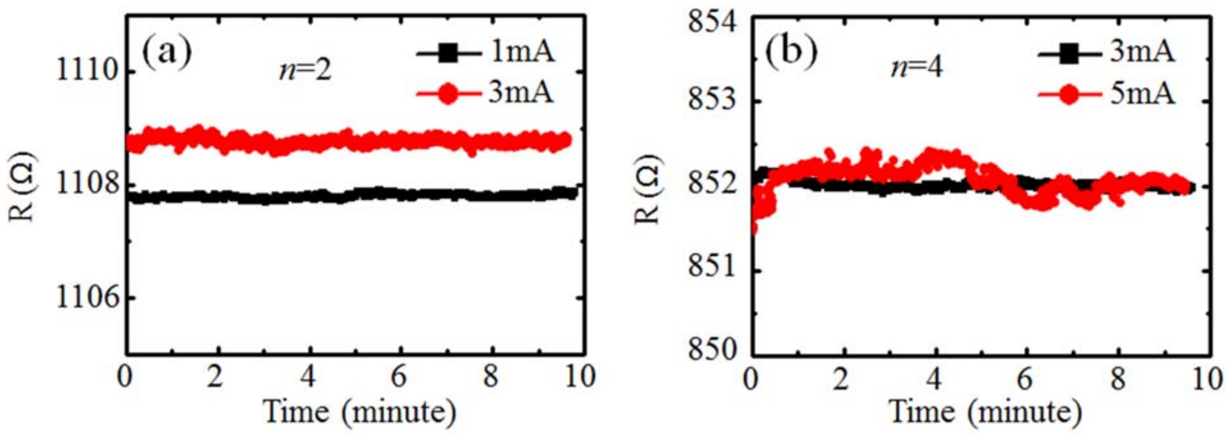

Figure S6. The wire resistance monitored by DC current flow for 10 minutes. (a) The wire resistance measured by 1mA and 3 mA for n = 2 structures. (b) The wire resistance measured by 3 mA and 5 mA for n = 4 structures.

For the n = 4 sample, no change in the wire resistance was observed as the DC bias was increased, as shown in Fig. S6(b). As such, the coercivity trend observed for n = 2 structures can be attributed to a combination of joule heating and SOT-induced switching.

S8. Effect of Oersted field on reversed domain nucleation

To show that the edge-defects do not play a role in the switching, the reversed process was repeated for a +z and −z initial magnetization. The reversed domains are nucleated at opposite current directions for +z and −z initial magnetizations, as shown in Figs. S7(a) and (b). Figure S7 shows that though the reversal occours from the edge, in both configurations, they occur at different locations, which is close to the current applied, implying that the defects do not play a role in the reversal domain nucleation.

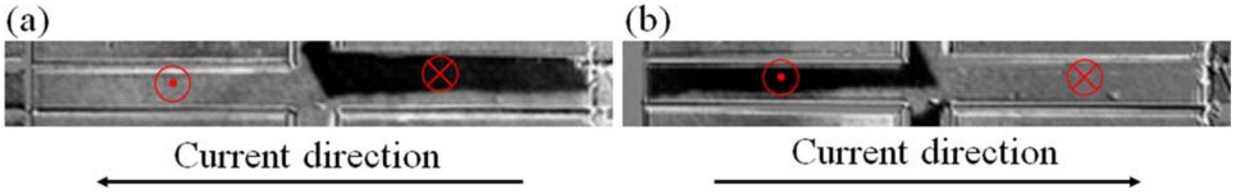

Figure S7. The reversal occurs from the edge for initial magnetization along (a) +*z* and (b) –*z* directions. The two configurations occurs at opposite current directions.

Reference

(1). Lee, H. R.; Lee, K.; Cho, J.; Choi, Y. H.; You, C. Y.; Jung, M. H.; Frédéric, B.; Shiota, Y.; Miwa, S.; Suzuki, Y. Spin-orbit torque in a bulk perpendicular magnetic anisotropy Pd/FePd/MgO system. Sci. Rep. **2014**, 4, 6548.

(2). Kawaguchi, M.; Moriyama, T.; Koyama, T.; Chiba, D.; Ono, T. Layer thickness dependence of current induced effective fields in ferromagnetic multilayers. J. Appl. Phys. **2015**, 117 (17), 17C730.

(3). Hayashi, M.; Kim J.; Yamanouchi, M.; Ohno, H. Quantitative characterization of the spin-orbit torque using harmonic Hall voltage measurements. Phys. Rev. B **2014**, 89 (14), 144425.

(4). Kim, J.; Sinha, J.; Hayashi, M.; Yamanouchi, M.; Fukami, S.; Suzuki, T.; Mitani, S.; Ohno, H. Layer thickness dependence of the current-induced effective field vector in Ta|CoFeB|MgO. Nat. Mater. **2013**, 12 (3), 240.

(5). Pi, U. H.; Kim, K. W.; Bae, J. Y.; Lee, S. C.; Cho, Y. J; Kim, K. S.; Seo, S. Tilting of the spin orientation induced by Rashba effect in ferromagnetic metal layer. Appl. Phys. Lett. 2010, **97** (16), 162507.

(6). Verhagen, T. G. A.; Leermakers, I.; van Ruitenbeek, J. M.; Aarts, J. Detecting Rashba fields at the interface between Co and Si oxide by ferromagnetic resonance. Phys. Rev. B 2015, **91** (18), 184402.
